# Computational multiqubit tunnelling in programmable quantum annealers

**DOI:** 10.1038/ncomms10327

**Published:** 2016-01-07

**Authors:** Sergio Boixo, Vadim N. Smelyanskiy, Alireza Shabani, Sergei V. Isakov, Mark Dykman, Vasil S. Denchev, Mohammad H. Amin, Anatoly Yu Smirnov, Masoud Mohseni, Hartmut Neven

**Affiliations:** 1 Google, Venice, California 90291, USA; 2 NASA Ames Research Center, Moffett Field, California 94035, USA; 3 Department of Physics and Astronomy, Michigan State University, East Lansing, Michigan 48824, USA; 4 D-Wave Systems Inc., Burnaby, British Columbia, Canada V5C 6G9; 5 Department of Physics, Simon Fraser University, Burnaby, British Columbia, Canada V5A 1S6

## Abstract

Quantum tunnelling is a phenomenon in which a quantum state traverses energy barriers higher than the energy of the state itself. Quantum tunnelling has been hypothesized as an advantageous physical resource for optimization in quantum annealing. However, computational multiqubit tunnelling has not yet been observed, and a theory of co-tunnelling under high- and low-frequency noises is lacking. Here we show that 8-qubit tunnelling plays a computational role in a currently available programmable quantum annealer. We devise a probe for tunnelling, a computational primitive where classical paths are trapped in a false minimum. In support of the design of quantum annealers we develop a nonperturbative theory of open quantum dynamics under realistic noise characteristics. This theory accurately predicts the rate of many-body dissipative quantum tunnelling subject to the polaron effect. Furthermore, we experimentally demonstrate that quantum tunnelling outperforms thermal hopping along classical paths for problems with up to 200 qubits containing the computational primitive.

## Abstract (web summary)

Quantum tunnelling may be advantageous for quantum annealing, but multiqubit tunnelling has not yet been observed or characterized theoretically. Here, the authors demonstrate that 8-qubit tunnelling plays a role in a D-Wave Two device through a nonperturbative theory and experimental data.

## Graphical abstract

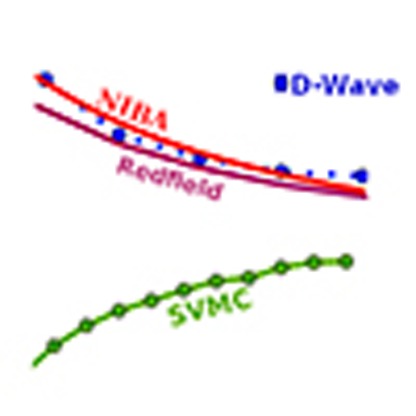

Quantum annealing^1^,^2^,^3^,^4^,^5^ is a technique inspired by classical simulated annealing^6^ that aims to take advantage of quantum tunnelling. In classical cooling optimization algorithms such as simulated annealing, the initial temperature must be high to overcome tall energy barriers. As the algorithm progresses, the temperature is gradually lowered to distinguish between local minima with small energy differences. This causes the stochastic process to freeze once the thermal energy is lower than the height of the barriers surrounding the state. In contrast, quantum tunnelling transitions are still present even at zero temperature. Therefore, for some energy landscapes, one might expect that quantum dynamical evolutions can converge to the global minimum faster than the corresponding classical cooling process.

The goal of quantum annealing is to find low-energy states of a ‘problem Hamiltonian'


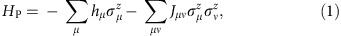


where the Pauli matrices 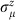 correspond to spin variables with values {±1}. The local fields {*h*_*μ*_} and couplings {*J*_*μν*_} define the problem instance. Quantum annealing is characterized by evolution under the Hamiltonian


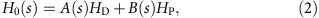


where 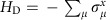. The annealing parameter *s* slowly increases from 0 to 1 throughout the annealing time *t*_*qa*_. Initially, 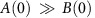. With increasing *s*, *A*(*s*) monotonically decreases to 0 for *s*=1, whereas *B*(*s*) increases.

The performance of chips designed to implement quantum annealing using superconducting electronics has been studied in a number of recent lines of work^7^,^8^,^9^,^10^,^11^,^12^,^13^,^14^,^15^,^16^,^17^,^18^,^19^,^20^,^21^,^22^,^23^,^24^. Given a quantum annealer operating at finite temperature, noise and dissipation strengths, does it utilize tunnelling or thermal activation for computation? Here we address precisely this question. We introduce a 16-qubit probe for tunnelling, a computational primitive where classical paths are trapped in a false minimum. We present a nonperturbative theory of multiqubit tunnelling, which takes into account both high- and low-frequency noises. To distinguish between tunnelling and thermal activation, we study the thermal dependence of the probability of success for the computational primitive. Thermal activation shows an increasing probability of success with increasing temperature, as expected. Multiqubit tunnelling, on the other hand, shows a decreasing probability of success with increasing temperature, both in theory and experiment. Finally, we study a generalization of the computational primitive to a larger number of qubits that contain the same ‘motif' multiple times. Quantum tunnelling outperforms thermal hoping for these problems under similar parameters.

## Results

### Quantum tunnelling probe

**Figure 1:**
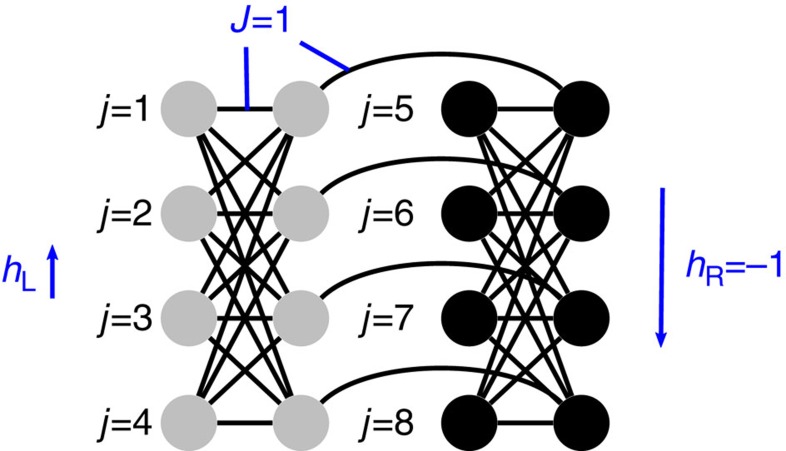
Graph of the tunnelling probe Hamiltonian. The 16 qubits are coupled ferromagnetically with *J*=1 (lines). The applied fields are 0<*h*_L_<*J*/2 (*h*_R_=−1) for the left (right) qubit cell. The symmetry and strong intracell ferromagnetic coupling makes each 8-qubit cluster evolve together.

We now describe a probe for computational tunnelling: a non-convex optimization problem consisting of just one global minimum and one false (local) minimum. For concreteness, we use the problem Hamiltonian depicted in Fig. 1, implementable in a D-Wave Two quantum annealer (a description of the flux qubits used in D-Wave Two is given in the Supplementary Note 1). The problem Hamiltonian consists of two cells, left and right, each with *n*=8 qubits. The local fields 0<*h*_L_<0.5 and *h*_R_=−1 are equal for all the spins within each cell, and all the couplings *J*=1 are ferromagnetic. The spins within each cell tend to move together as clusters because of symmetry and the strong intracell ferromagnetic coupling energy. We choose |*h*_R_|>|*h*_L_| so that in the low-energy states of *H*_P_ the right cluster is pointing along its own local field as seen in Fig. 1. The difference in energy of the states with opposite polarization in the left cluster is *n*(*J*−2*h*_L_). Choosing *h*_L_<*J*/2=0.5, the global minimum corresponds to both clusters having the same orientation, while in the false minimum they have opposite orientations.

**Figure 2:**
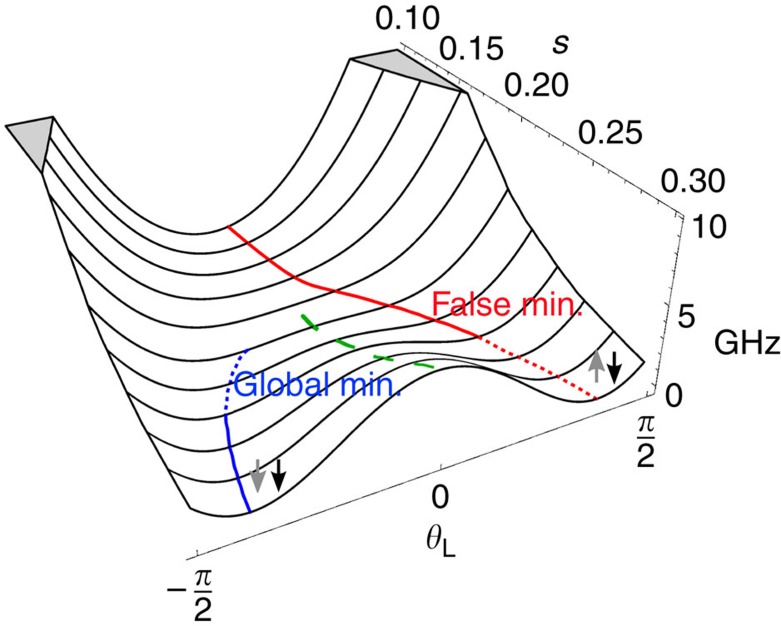
Effective energy potential using *h*_L_=0.44. The mean field potential is plotted versus annealing parameter *s* and tilt angle *θ*_L_ of each spin vector in the left cluster. The red line corresponds to a path that starts in the initial global minimum and follows the instantaneous local energy minimum. A second local minimum (dashed blue line) forms at the bifurcation point *s*=0.18. The global minimum is found in this second path after *s*=0.24 (dashed to continuous blue line). To reach this global minimum the system state has to traverse the energy barrier between them (dashed green line), either by thermal activation or by quantum tunnelling.

We can gain an intuitive understanding of the effective energy landscape if we represent each qubit by a mean field spin vector in the *xz* plane. Denote by *θ*_*μ*_ the angle of the spin vector for qubit *μ* with the *x* quantization axis. We assume that all the qubits in the left (right) cluster have the same angle *θ*_L_ (*θ*_R_). This assumption is based on symmetry and the strong intracluster ferromagnetic energy. The resulting energy potential can also be derived using more formal methods, such as the Villain representation (see Supplementary Note 2 and ref. 25). Figure 2 plots the effective energy potential for the left cluster as a function of *θ*_L_ with *h*_L_=0.44. At the beginning of the annealing process 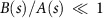, and we have 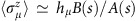 (the coupling terms are quadratic in the z polarizations 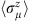 and therefore negligible at this point). As *h*_L_ and *h*_R_ have opposite signs, so will the *z-*projections of spins in the two clusters early in the evolution. To escape this path classically all spins in the left cluster must flip sign, which requires traversing an energy barrier. The barrier peak corresponds to zero total *z*-polarization of the left cluster. Therefore, the barrier grows with the ferromagnetic energy of the cluster (*n*/2)^2^*J*. The barrier height is much greater than the residual energy, which grows with *n*(*J*−2*h*_L_).

**Figure 3:**
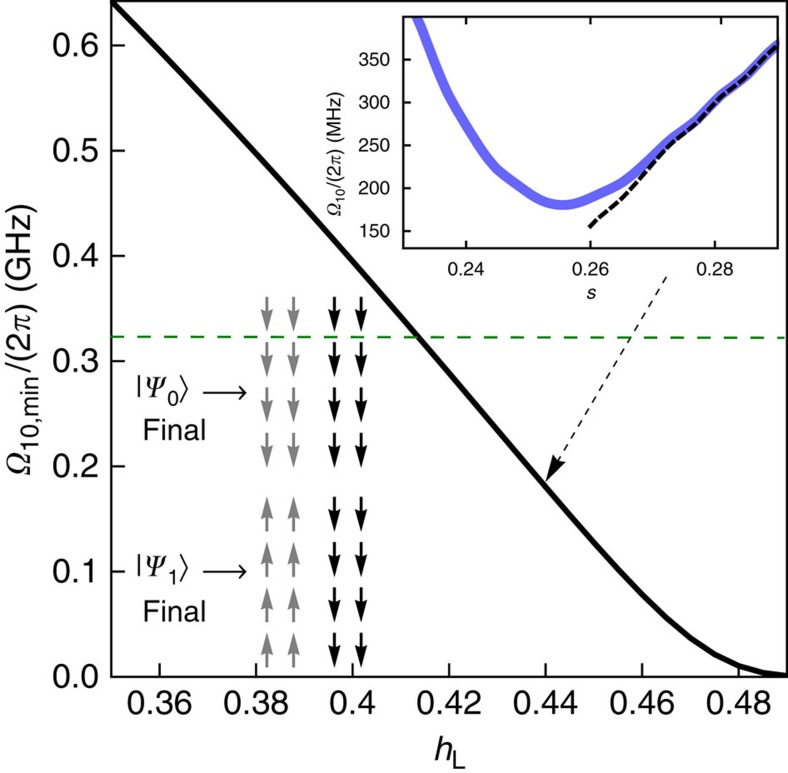
Quantum energy gap. Inset shows the gap *ħΩ*_10_=*E*_1_(*s*)−*E*_0_(*s*) versus *s*, using *h*_L_=0.44. The dashed line is the gap in the diabatic (pointer) basis. In the main plot, the minimum gap decreases with *h*_L_. The horizontal green dashed line (324 MHz) corresponds to 15.5 mK, the lowest temperature in our experiments. The lower inset shows the spin configurations of the two lowest eigenstates at the end of the annealing.

Quantum mechanically, if *h*_*L*_<*J*/2 the system evolution goes through an ‘avoided-crossing' where the two lowest eigenstates *E*_1_(*s*) and *E*_0_(*s*) approach closely to, and then repel from, each other (see inset in Fig. 3). Higher-energy states remain well separated during the evolution. This level repulsion occurs because of the collective tunnelling of qubits in the left cluster between the opposite *z* polarizations. At the point where the gap *ħΩ*_10_(*s*)=*E*_1_(*s*)−*E*_0_(*s*) reaches its minimum, the corresponding adiabatic eigenstates are formed by the symmetric and antisymmetric superpositions of the cluster orientations. The size of the minimum gap varies with *h*_L_ as seen in Fig. 3. The position of the avoided-crossing can be estimated to occur at the point where 
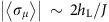
 and moves towards *s*=1 as *h*_L_ approaches *J*/2. Note that for *h*_L_=*J*/2 the residual energy *n*(*J*−2*h*_L_) vanishes and the final ground space is degenerate. There is no avoided-crossing when *h*_L_>*J*/2 (Supplementary Fig. 1).

### Characterization of noise and dissipation

Under realistic conditions, a quantum annealer can be strongly influenced by coupling to the environment. We introduce a detailed phenomenological open quantum system model based on single-qubit measurable noise parameters. We shall assume that each qubit is coupled to its own environment with an independent noise source. In the concrete case studied here, this is consistent with experimental data^9^, and the coupling of the environment to each flux qubit is proportional to a *σ*^*z*^ qubit operator (flux fluctuations).

In the analysis of the transitions between the states we start from the initial (gapped) stage when the instantaneous energy gap *ħΩ*_10_(*s*) between the two lowest eigenstates 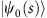, 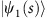 is sufficiently large compared with the linewidth *ħW*. Then, the coupling to the environment can be treated as a perturbation and the transition rate between these states is given by Fermi's golden rule 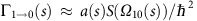, where *S*(*ω*) is the noise spectral density (see Methods). Here


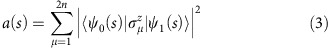


is a sum of (squared) transition matrix elements between the two eigenstates.

**Figure 4:**
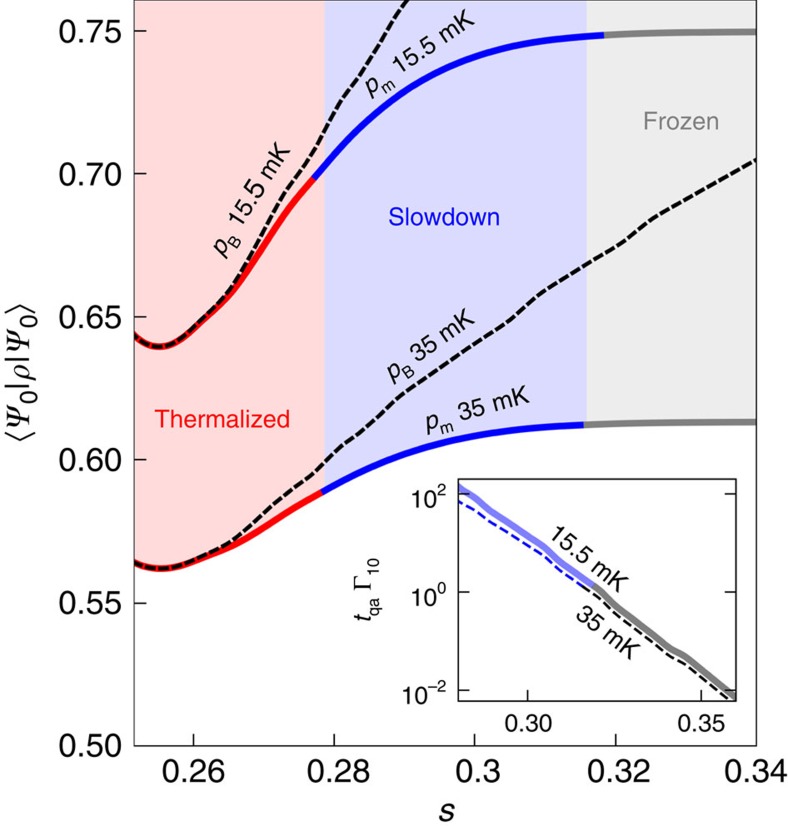
Multiqubit freezing. Solid lines correspond to the modelled population *p*_m_ of the lowest-energy eigenstate along the quantum annealing process using *h*_L_=0.44 at 15.5 mK (top line) and 35 mK (bottom line). Dashed lines correspond to the thermal equilibrium population *p*_*B*_. In the thermalization phase (red) the transition rate is fast and the population remains close to thermal equilibrium. As the multiqubit energy barrier increases, the transition rates are exponentially reduced with *s*, as shown in the inset. We define the slowdown regime (blue) as *t*_*qa*_Γ_1→0_<10 and the frozen regime (grey) as *t*_*qa*_Γ_1→0_<0.1. Comparing the data at 15.5 and 35 mK, we see a small change in the transition rate relative to the larger change in the thermal equilibrium ground-state population. Therefore, the probability of success is lower at higher temperature.

In the minimum gap region, the (squared) matrix element *a*(*s*) for the transition rate is large, and the system is thermalized (Fig. 4). More precisely, we have 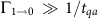, where the inverse of the annealing time 1/*t*_*qa*_ is an approximation for the annealing rate. The ground-state population is given by the Boltzmann distribution at the experimental temperature.

After the avoided-crossing region (at *s*=0.255) we observe a steep exponential fall-off of the matrix element *a*(*s*) with *s*, eventually causing multiqubit freezing (Fig. 4). Multiqubit freezing is quite distinct from single-qubit freezing. Single-qubit tunnelling^10^ decays slowly as the magnitude of the transverse field *A*(*s*) decreases. The multiqubit transition rate, however, decays exponentially fast (see inset of Fig. 4). This is due to the increasing effective barrier width (Fig. 2), which results in an exponential decrease in quantum tunnelling and a slowdown of the transition rate Γ_1→0_. Formally, the barrier width corresponds to the Hamming distance


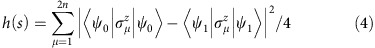


between the opposite *z* orientations of the left cluster in the two lowest-energy eigenstates. The exponential sensitivity of multiqubit tunnelling to the width or Hamming distance *h*(*s*) is the cause of the exponential decay of the matrix element *a*(*s*), and of the multiqubit freezing.

We distinguish a slowdown phase (roughly, 0.1<*t*_*qa*_Γ_1→0_<10) and a frozen phase (*t*_*qa*_Γ_1→0_<0.1). In the frozen phase, there are no dynamics. Part of the system population remains trapped in the excited state 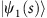 corresponding to the false minimum of the effective potential until the end of the quantum annealing process (Fig. 4).

When the energy gap is similar to (or smaller than) the noise linewidth *W*, the environment cannot be treated as a perturbation. We develop a multiqubit nonperturbative analysis in the spirit of the Non-interacting Blip Approximation (NIBA)^26^ that covers all quantum annealing (QA) stages. In the slowdown phase, when the Hamming distance approaches its maximum value, *h*∼*n*, the instantaneous decay rate of the first excited state takes the form


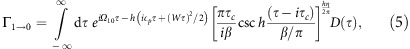


where *W* and 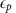 are the linewidth and reconfiguration energy from the low-frequency noise, *η* and 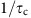 are the high-frequency Ohmic noise coupling and cutoff and *β* is the inverse temperature *ħ*/*k*_*B*_*T*. The dependence on the annealing parameter *s* is implicit. The factor 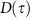 is related to the tunnelling permeability of the potential barrier in Fig. 2 (similar to the coefficient *a*). It has the expression


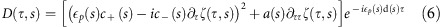


where


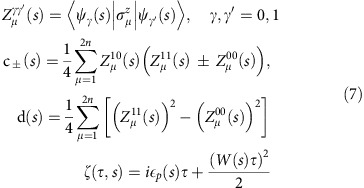


Equation (5) describes collective tunnelling of the left qubit cluster assisted by the environment. The crucial difference from the single-qubit theory^27^,^28^ is that the parameters of the environment in the transition rate are rescaled by the barrier width or Hamming distance *h*(*s*). The effective low-frequency noise linewidth is *h*^1/2^(*s*)*W*(s), the reconfiguration energy is 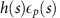 and the Ohmic coefficient is *h*(*s*)*η*(*s*). This is important at the late stages of quantum annealing when 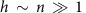.

### Comparison of NIBA with data

**Figure 5:**
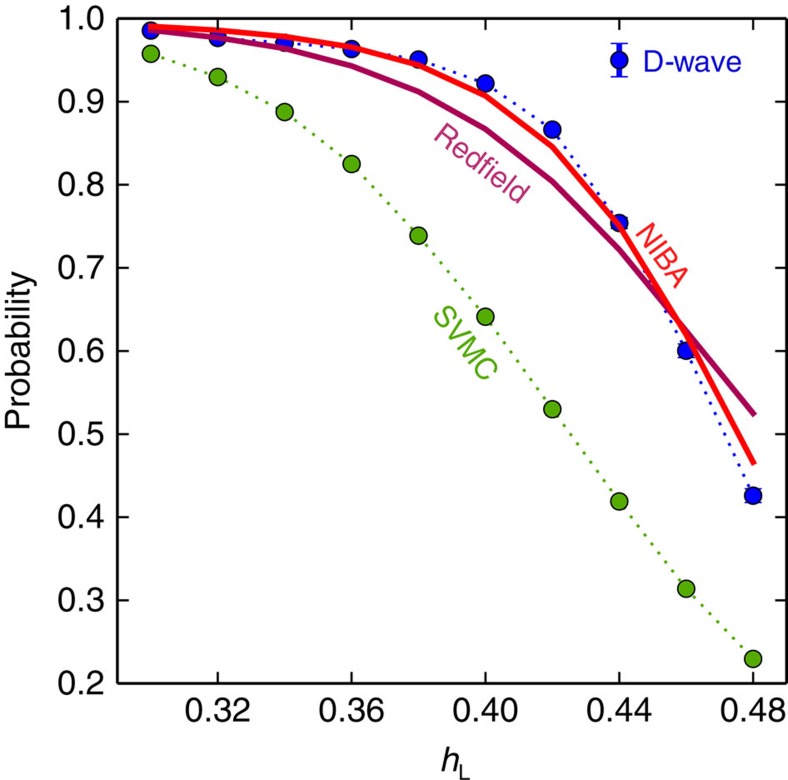
Probability of success versus *h*_L_. We plot the probability of measuring the final ground state for different values of *h*_L_. The physical temperature for D-Wave is 15.(5) mK, and the annealing time is 20 μs. Both Redfield and NIBA use only measured parameters (no fitting). SVMC uses an algorithmic temperature equal to the physical temperature and 128,000 sweeps, as explained in the text. Error bars (s.e.m.) are smaller than markers.

**Figure 6:**
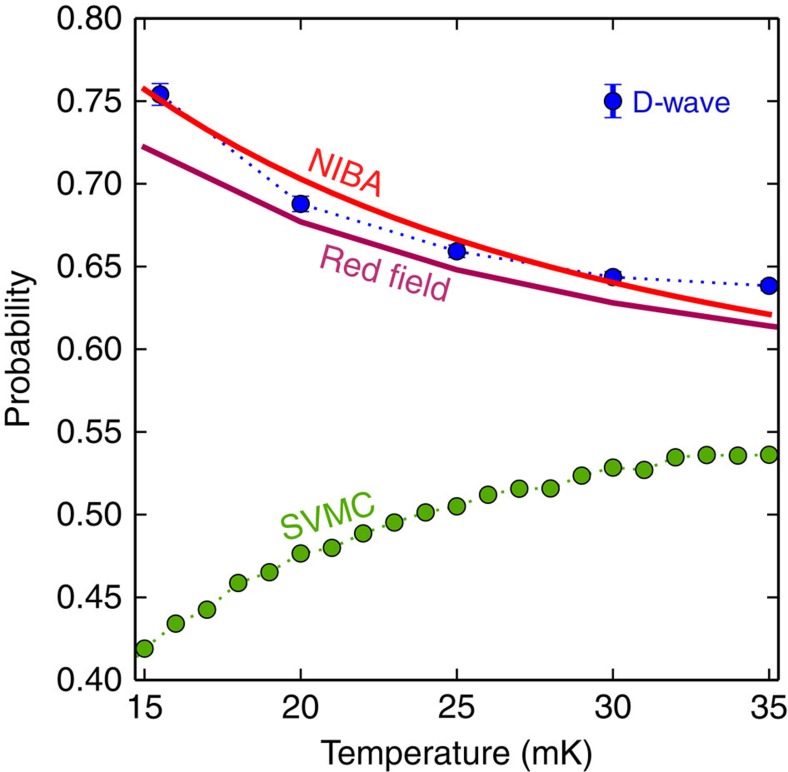
Probability of success versus temperature for *h*_L_=0.44. The decreasing probability with increasing temperature is only matched with theories based on quantum tunnelling. This is the opposite tendency to thermal activation (SVMC). The annealing time is 20 μs. Both Redfield and NIBA use only measured parameters (no fitting). SVMC uses an algorithmic temperature equal to the physical temperature and 128,000 sweeps. In this temperature range the lowest two states (the double-well potential) account for all the probability (0.9998 for D-Wave, 0.99998 for SVMC). Error bars (s.e.m.) are smaller than markers.

We observe a very close correspondence between the results of the analysis with the NIBA Quantum Master Equation for the dressed cluster states and the D-Wave Two data displayed in Fig. 5, which shows the dependence of the ground-state population on *h*_L_. We emphasize that for NIBA (and the standard Redfield equation with Ohmic *S*_oh_(*ω*), see Methods) we do not have any parameter fitting: the parameters are obtained from experiments (see Methods). The success probability of quantum annealing is (roughly) determined by the thermal equilibrium ground-state population during the slowdown phase (Fig. 4). When the temperature grows, the ground-state population decreases appreciably, while the transition rate changes little. Consequently, quantum mechanically, the probability of success decreases with increasing temperature, as seen in Fig. 6, for sufficiently big gaps. Figure 6 shows the dependence of the ground-state population with temperature.

For *h*_L_ closer to the degeneracy value *h*_L_=*J*/2, the minimum gap 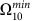 becomes smaller, as seen in Fig. 3. Where 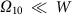, the adiabatic basis of the instantaneous multiqubit states 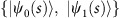 loses its physical significance. Because the coupling to the bath is relatively strong here, the system quickly approaches the states corresponding to predominantly opposite cluster orientations, similar to diabatic states (see inset of Fig. 3). Transitions between these states, also called pointer states^29^, occur at a much slower rate as a consequence of the polaronic effect. As a result, for sufficiently small mininum gaps the multiqubit freezing starts before the avoided-crossing and the success probability increases with temperature^12^. This is captured by the multiqubit NIBA equation, but not by the standard Redfield Quantum Master Equation.

### Spin vector Monte Carlo

We want to distinguish quantum tunnelling from thermal activation along classical paths of product states (which preclude multiqubit tunnelling). To give a more precise description of the classical paths of product states, let each qubit be represented by a mean field spin vector in the *xz* plane and denote by *θ*_*μ*_ the angle of the spin vector for qubit *μ* with the *x* quantization axis, as before. A classical path (red line in Fig. 2) that follows the local minimum of the effective energy potential gets trapped in a false minimum and fails to solve the corresponding optimization problem, as explained above. In the absence of quantum tunnelling, the global minimum could be reached through thermal excitations for over-the-barrier escape from the false minimum. This thermal activation results in an increasing probability of success with rising temperature.

This intuition is supported by spin vector Monte Carlo (SVMC), a numerical algorithm consisting of thermal Metropolis updates of the spin vectors. SVMC was introduced recently in ref. 20 and studied in related lines of work^21^,^23^. The dynamics are constrained to spin–vector product states, with one spin vector per qubit. For a given Hamiltonian *H*_0_(*s*), we denote the corresponding energy by *E*_*s*_(*θ*_1_,…,*θn*_q_), where *n*_q_ is the number of qubits. The evolution consists of a sequence of sweeps along the Hamiltonian path {*H*_0_(*s*)}. In each sweep, a Monte Carlo update is proposed for each qubit in two steps. First, a new angle 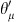
is drawn from the uniform distribution in [0, 2*π*]. Second, the spin vector for qubit *μ* is updated 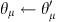
according to the Metropolis rule for the energy difference


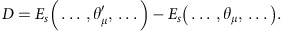


That is, the move is always accepted if *D* is negative, and with probability given by the Boltzmann factor exp(−*D*/*k*_*B*_*T*) if *D* is positive.

**Figure 7:**
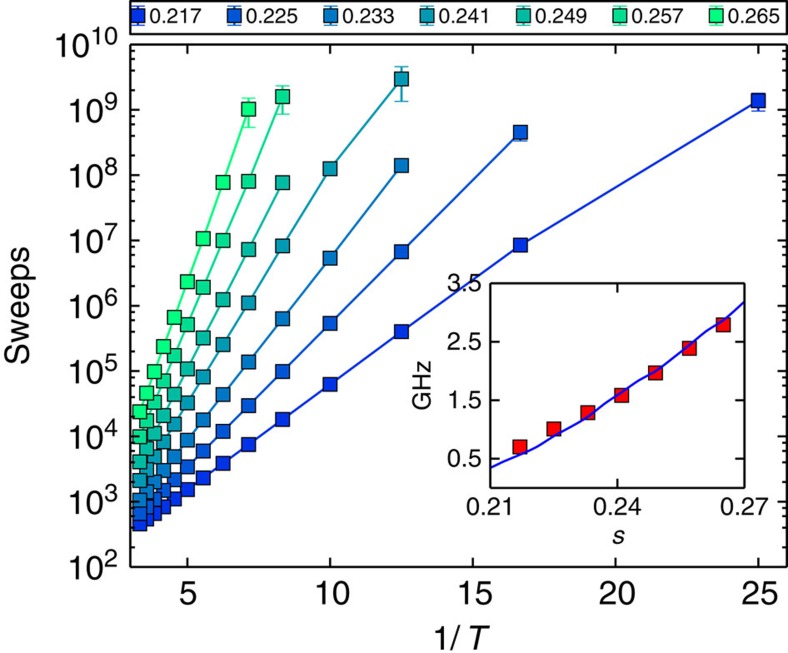
Analysis of the activation energy for Kramer's scape for SVMC. The main figure shows, in a semilog scale, the average number of sweeps as a function of temperature. We plot lines for different points in the annealing schedule, from *s*=0.217 (dark blue, see legend) to *s*=0.265 (green). Error bars correspond to the s.e.m. The embedded figure shows the activation energy (red dots) and the semiclassical energy barrier (blue). There is a good correspondence between SVMC and the effective energy potential.

The initial state is chosen to be the global minimum of the transverse field. For low *T* and sufficient sweeps, the evolution proceeds along the false minima path of Fig. 2. This numerical method allows us to study thermal hopping between the classical paths. To check this correspondence, we studied the height of the energy barrier obtained from Kramers' theory applied to SVMC. For the effective potential at a fixed value of *s*, we initialized the spin vector state at a local minima, and watch for Kramers events. A Kramers event corresponds to the arrival at the other minima under thermal activation. According to Kramers' theory, the dependence on temperature for the expected number of sweeps necessary for a Kramers event follows the formula exp(Δ*U*/*T*), where Δ*U* is the height of the energy barrier. We extract the energy barrier by fitting the curve of the average number of sweeps for different *T*. We find that this matches almost exactly the energy barrier height in Fig. 2 for different values of *s* (Fig. 7).

**Figure 8:**
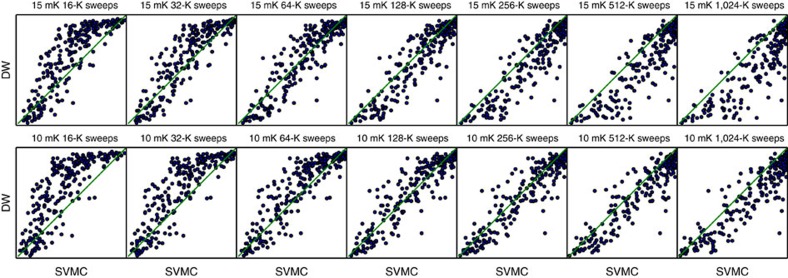
Scatter plots showing the correlation of D-Wave Two data and SVMC. Correlations for the random Ising benchmark for different algorithmic temperatures (in mK) and number of sweeps. We will use the parameters *T*=15 mK and sweeps=128,000 for SVMC in the rest of the paper.

A disadvantage of SVMC as outlined above and introduced in ref. 20 is that there is no natural choice to relate the number of sweeps to the physical evolution time. As in other lines of work, we will choose the number of sweeps to obtain a good correlation with the probability of success of the D-Wave chip for a benchmark of random Ising models with binary couplings *J*_*μν*_∈{1, −1} (refs 15, 18, 20, 23). This will allow us to phenomenologically correlate the number of sweeps to physical time. We set the algorithmic temperature of SVMC to be the same as the physical temperature because we are interested in the dependence of the success probability with temperature. There are no important differences for the correlation with other temperature choices. The correlation with the random Ising benchmark for 128,000 sweeps (Fig. 8) is 0.92, and the residual probabilities *P*_SVMC_−*P*_D−Wave_ have a mean of 0.05 and a s.d. of 0.12. This is consistent with the best values found over a wide range of parameters. We therefore use 128,000 sweeps at 15 mK as our reference parameters for SVMC.

Figure 6 confirms the thermal activation in SVMC. This is opposite to both open quantum system theory and experiments with the D-Wave chip, which show a reduction in the probability of success with rising temperature, as explained above. Furthermore, Fig. 5 shows that the probability of success for SVMC is lower than the probability of success for D-Wave and open system quantum models.

### Larger problems

**Figure 9:**
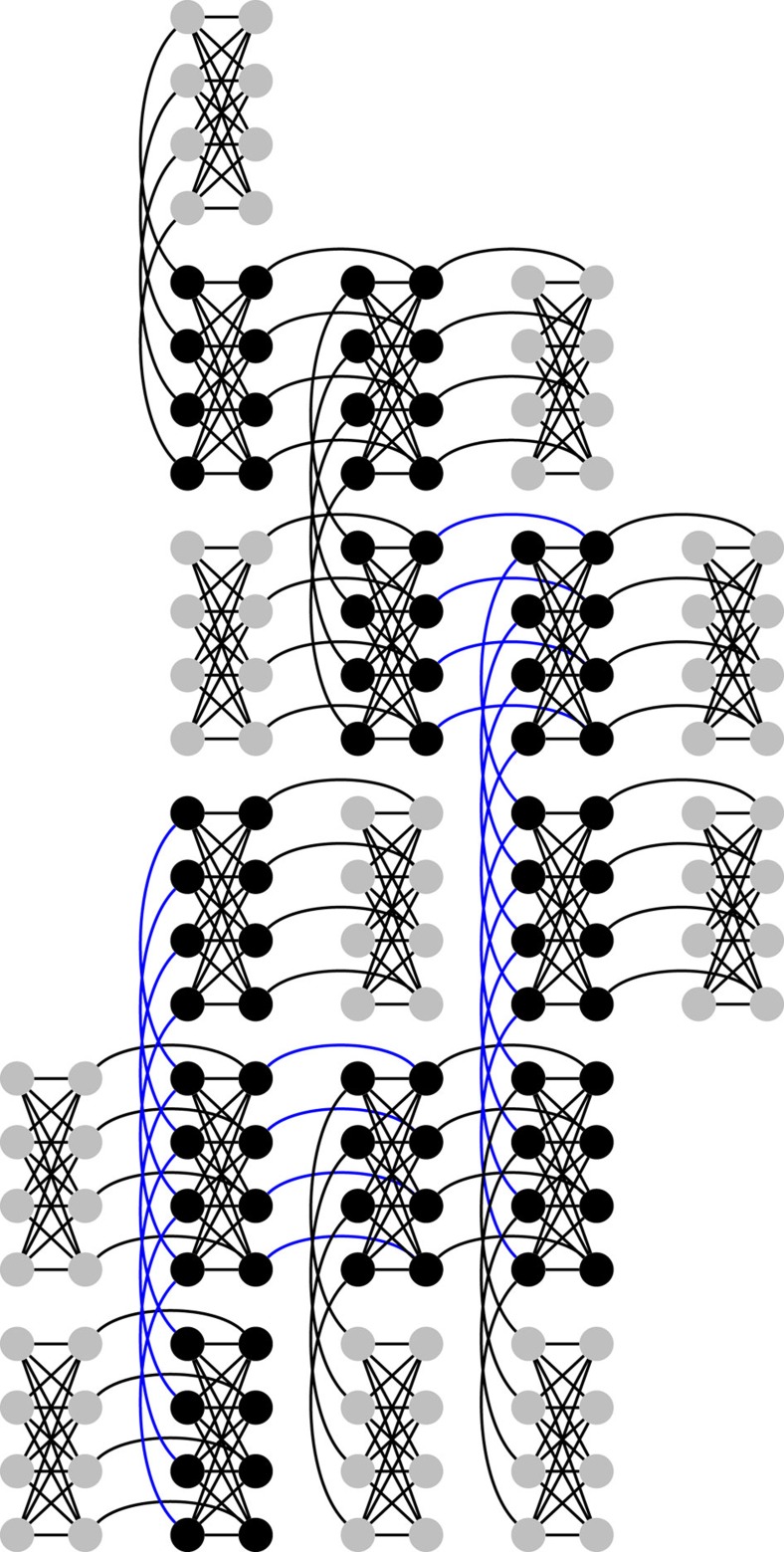
Larger problems that contain the tunnelling probe ‘motif' as subproblems. As in Fig. 1, the black (grey) cluster has a strong *h*_R_=−1 (weak *h*_L_=0.44) local field. The black clusters are connected in a glassy manner to make the problem less regular: all connections between any two neighbouring black clusters are set randomly to either −1 or +1. The −1 connections are depicted in blue.

**Figure 10:**
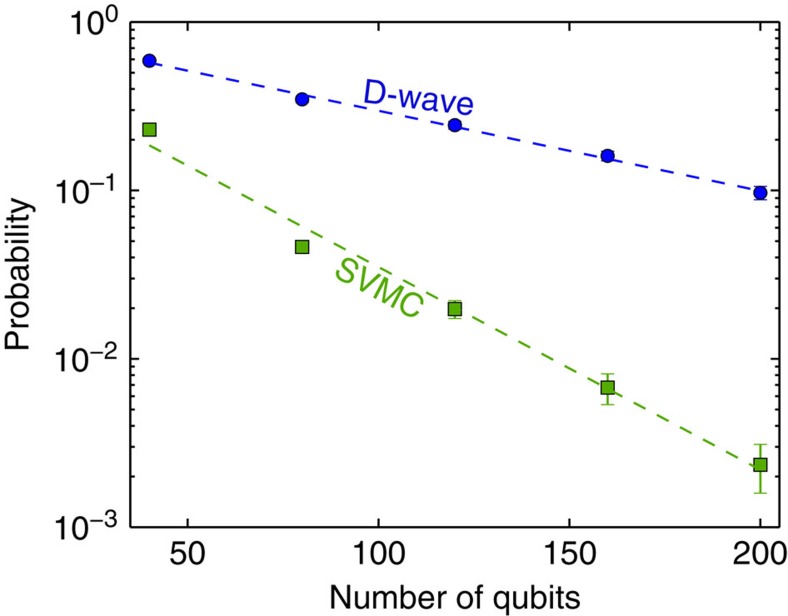
Success probability for a glass of clusters as a function of the number of qubits. We fit the mean probability of success *P*(*n*_q_)∝exp(−*αn*_q_) as a function of the number of qubits *n*_q_ (dashed lines). The fitting exponent *α* for the D-Wave Two data is (1.1±0.05) × 10^−2^, while the fitting exponent for the SVMC numerics is (2.8±0.17) × 10^−2^. The error estimates for the exponents (s.e.m.) are obtained by bootstrapping.

A generalization of the 16-qubit problem to a larger number of qubits is achieved by studying problems that contain the same ‘motif' (Fig. 1) multiple times within the connectivity graph (Fig. 9). The success probabilities for up to 200 qubits are shown in Fig. 10. We fit the average success probability as *P*(*n*_q_)∝exp(−*αn*_q_), where *n*_q_ is the number of qubits. The fitting exponent *α* for the D-Wave Two data is (1.1±0.05) × 10^−2^, while the fitting exponent for the SVMC numerics is (2.8±0.17) × 10^−2^. We conclude that, for instance with multiqubit quantum tunnelling, the D-Wave Two processor returns the solution that minimizes the energy with consistently higher probability than physically plausible models of the hardware that only employ product states and do not allow for multiqubit tunnelling transitions.

## Discussion

The role of multiqubit tunnelling as a computational resource is an open problem of active research. Nevertheless, it is instructive to consider some plausible estimates for the case of minimization of an Ising problem that contains pairwise interactions between all qubits. A way to think of multiqubit tunnelling as a computational resource is to regard it as a form of large neighbourhood search. Collective tunnelling transitions involving *K* qubits explore a *K* variable neighbourhood, and there is a combinatorial number of such neighbourhoods. Using standard resources, the cost of exhaustively searching on a Hamming ball of binary strings of radius *K* is 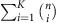, which is bounded from below by 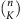, where *n* is the total number of qubits. Therefore, for 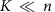, the cost is 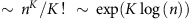. As one can see, the exponent in *K* is very steep (log *n*). On the other hand, for *K*∼*n*/2 the exponent is that of the exhaustive search in the entire *n*—bit string space (2^*n*^). We now compare this with the tunnelling rate across a barrier of width *K*. It is given by exp(−*cK*) for some constant *c*. If *K* is smaller than *n* then, as *n* increases, 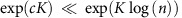 and tunnelling can be faster than large neighbourhood search. On the other hand, in many problems similar to the two-cluster problem the barrier width is *K* in *O*(*n*). In these cases the tunnelling rate can still be 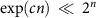 (refs 30, 31, 32, 33, 34, 35). Therefore, again tunnelling can still provide a dramatically faster search option.

We find that the current-generation D-Wave Two annealer enables tunnelling transitions involving at least 8 qubits. It will be an important future task to determine the maximal *K* attainable by current technology and how large it can be made in next generations. The multiqubit polaronic quantum master equation presented here lays down the theory to answer this question. It guides the design of next-generation architectures and helps to understand for which computational problems quantum-enhanced optimization may offer an advantage. The larger the *K* the easier it should be to translate the quantum resource ‘*K*-qubit tunnelling' into a possible computational speedup. We want to emphasize that this paper does not claim to have established a quantum speedup. To this end, one would have to demonstrate that no known classical algorithm finds the optimal solution as fast as the quantum process. To establish such an advantage it will be important to study to what degree collective tunnelling can be emulated in classical algorithms such as Quantum Monte Carlo or by employing cluster update methods. However, the collective tunnelling phenomena demonstrated here present an important step towards what we would like to call a physical speedup: a speedup relative to a hypothetical version of the hardware operated under the laws of classical physics.

## Methods

### Experimental properties of the noise

The properties of the noise are determined by the noise spectral density *S*(*ω*), which is characterized by single-qubit macroscopic resonant tunnelling (MRT) experiments in a broad range of biases (0.4 MHz–4 GHz) and temperatures (21–38 mK) for tunnelling amplitudes of a single flux qubit below 1 MHz. The MRT data collected are surprisingly well described^28^,^36^ by a phenomenological ‘hybrid' thermal noise model *S*(*ω*)=*S*_lf_(*ω*)+*S*_oh_(*ω*). Here 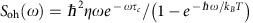 denotes the high-frequency part and has Ohmic form with dimensionless coupling *η* and cutoff frequency 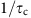 (assumed to be very large). The low-frequency part *S*_lf_ is of the 1/*f* type^37^, and in current D-Wave chips this noise is coupled to the flux qubit relatively strongly. Its effect can be described with only two parameters: the width *W* and the Stokes shift 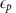 of the MRT line^27^. The experimental shift value is related to the width by the fluctuation-dissipation theorem 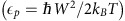 and represents the reorganization energy of the environment. The values of the noise parameters measured at the end of the annealing (*s*=1) for the D-Wave Two chip are *W*/(2*π*)=0.40(1) GHz and *η*=0.24(3).

## Additional information

**How to cite this article:** Boixo, S. *et al.* Computational multiqubit tunnelling in programmable quantum annealers. *Nat. Commun.* 7:10327 doi: 10.1038/ncomms10327 (2016).

## Supplementary Material

Supplementary Information Supplementary Figures 1-5, Supplementary Notes 1-2 and Supplementary References.
