# Supplementary material for: Computational multiqubit tunnelling in programmable quantum annealers
Source: Nat Commun. 2016 Jan 7;7:10327. doi: 10.1038/ncomms10327 (PMC4729842; doi:10.1038/ncomms10327)
Supplement: Supplementary Information — Supplementary Figures 1-5, Supplementary Notes 1-2 and Supplementary References. [file ncomms10327-s1.pdf]

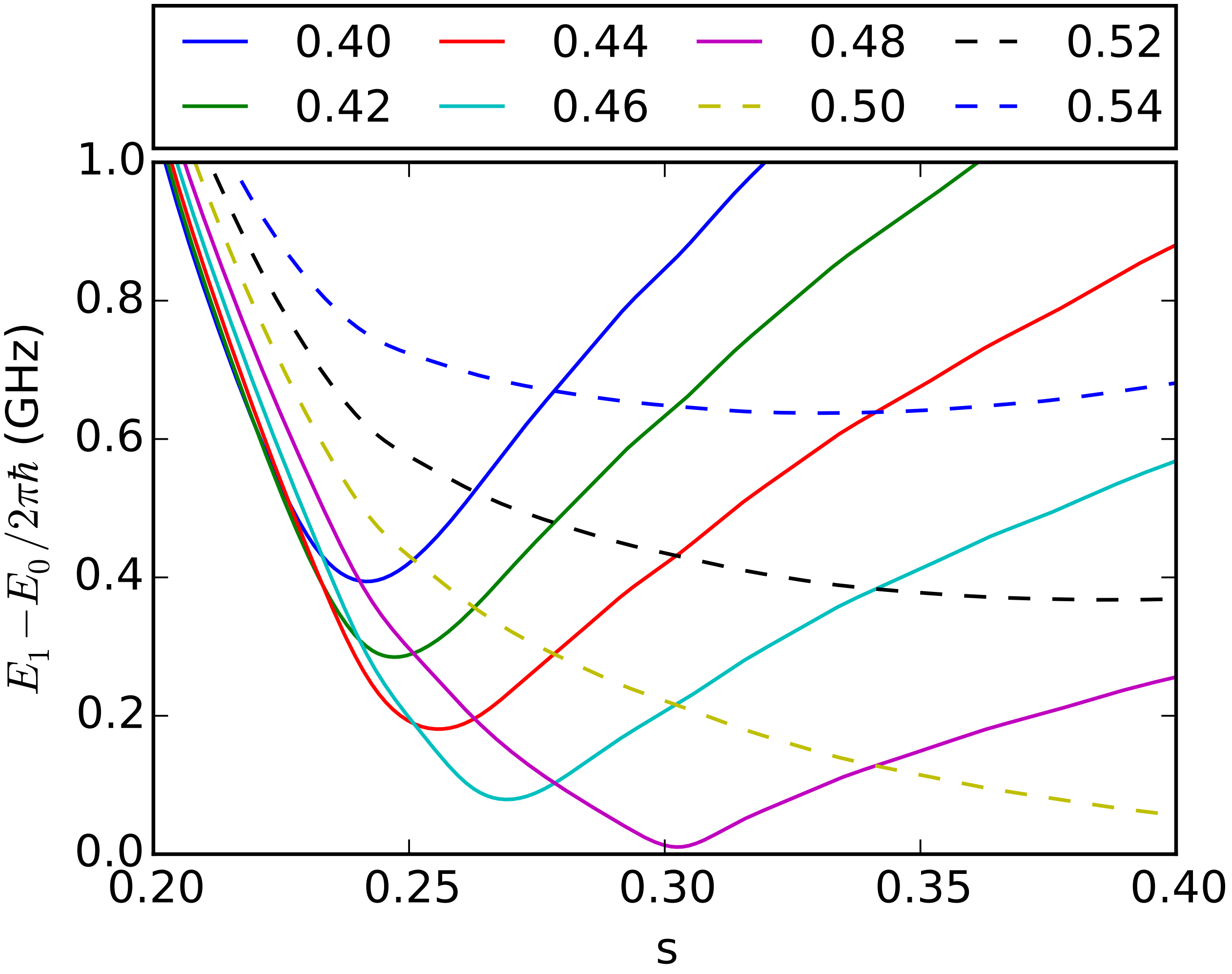

Supplementary Figure 1: **Gap for different values of h/J.** Plot of the gap as a function of the annealing parameter s for different values of h/J (see lagend). For $h < 2J$ (continuous lines) there is an avoided crossing. For $h > 2J$ there is no avoided crossing. At $h = 2J$ the final ground space is degenerate

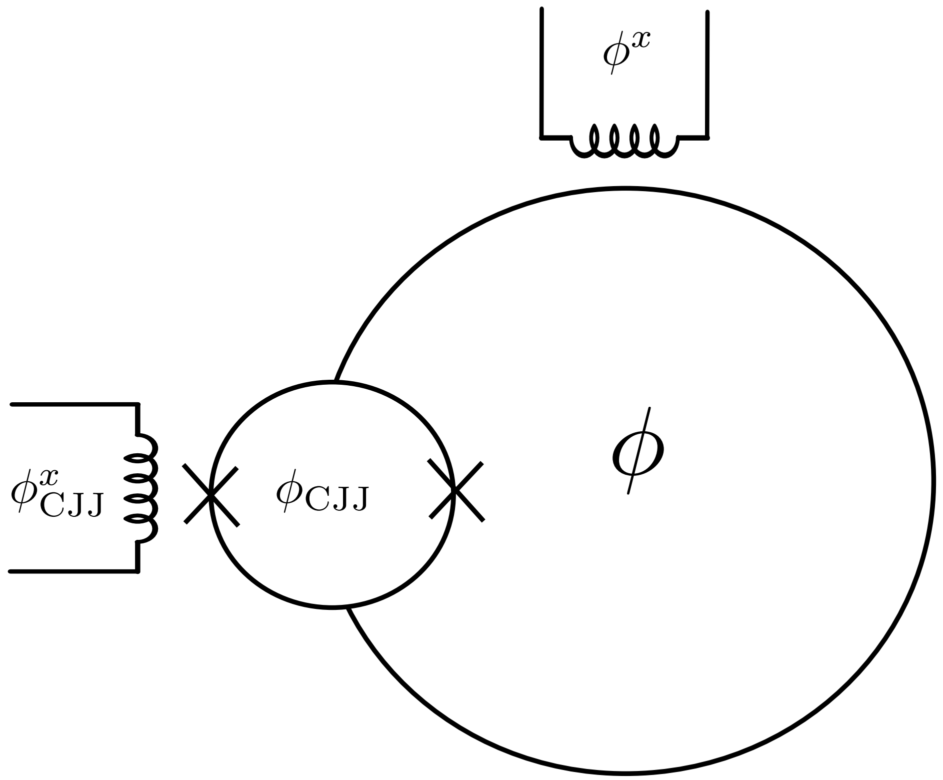

Supplementary Figure 2: **Schematic of a Compound Josephson Junction (CJJ) qubit.**

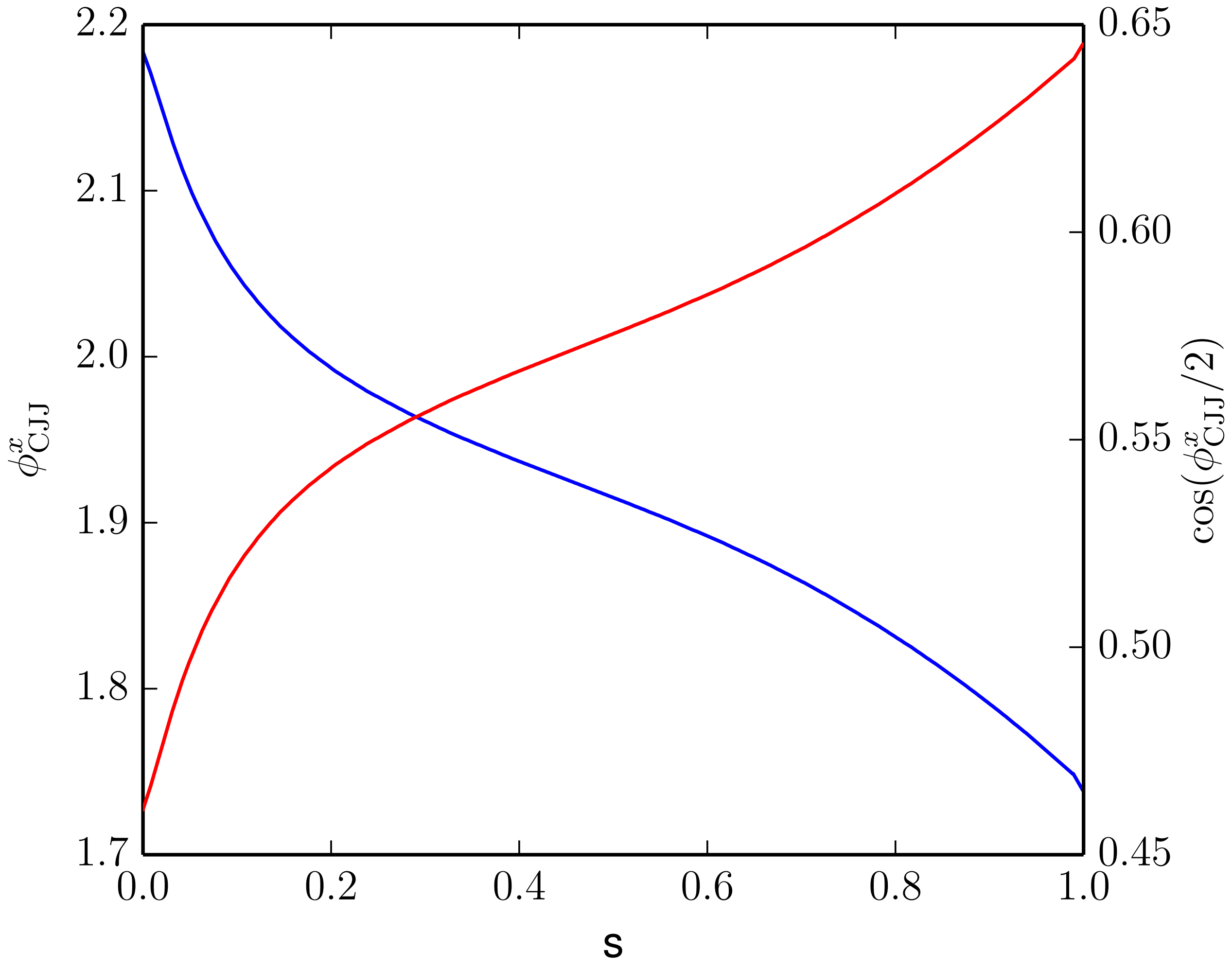

Supplementary Figure 3: **The CJJ external phase $\phi^x_{\rm CJJ}$ (blue) and $\cos(\phi^x_{\rm CJJ})$ (red).** The dependence of $\phi^x_{\rm CJJ}$ on the parameter s is chosen so that the persistent current $I_p(s)$ scales linearly with s, see Eq. 12.

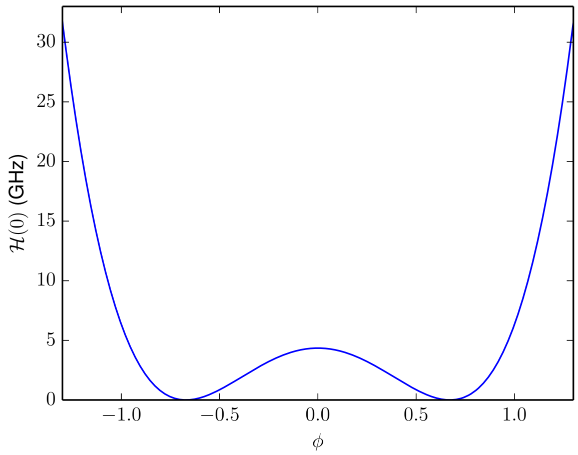

(a) Simplified 1D potential.

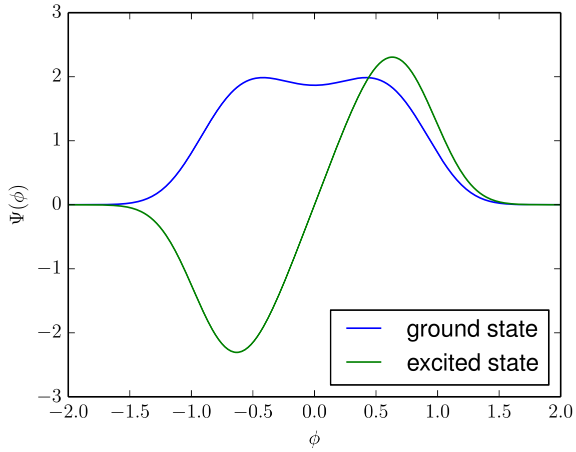

(b) First two eigenstates.

Supplementary Figure 4: **Flux qubit potential.** (a) The simplified 1D potential $\mathcal{H}(\phi^x = 0)$ of Eq. 2 for annealing parameter $s = 0.278$. (b) The first two eigenvectors of the flux qubit potential for $s = 0.278$.

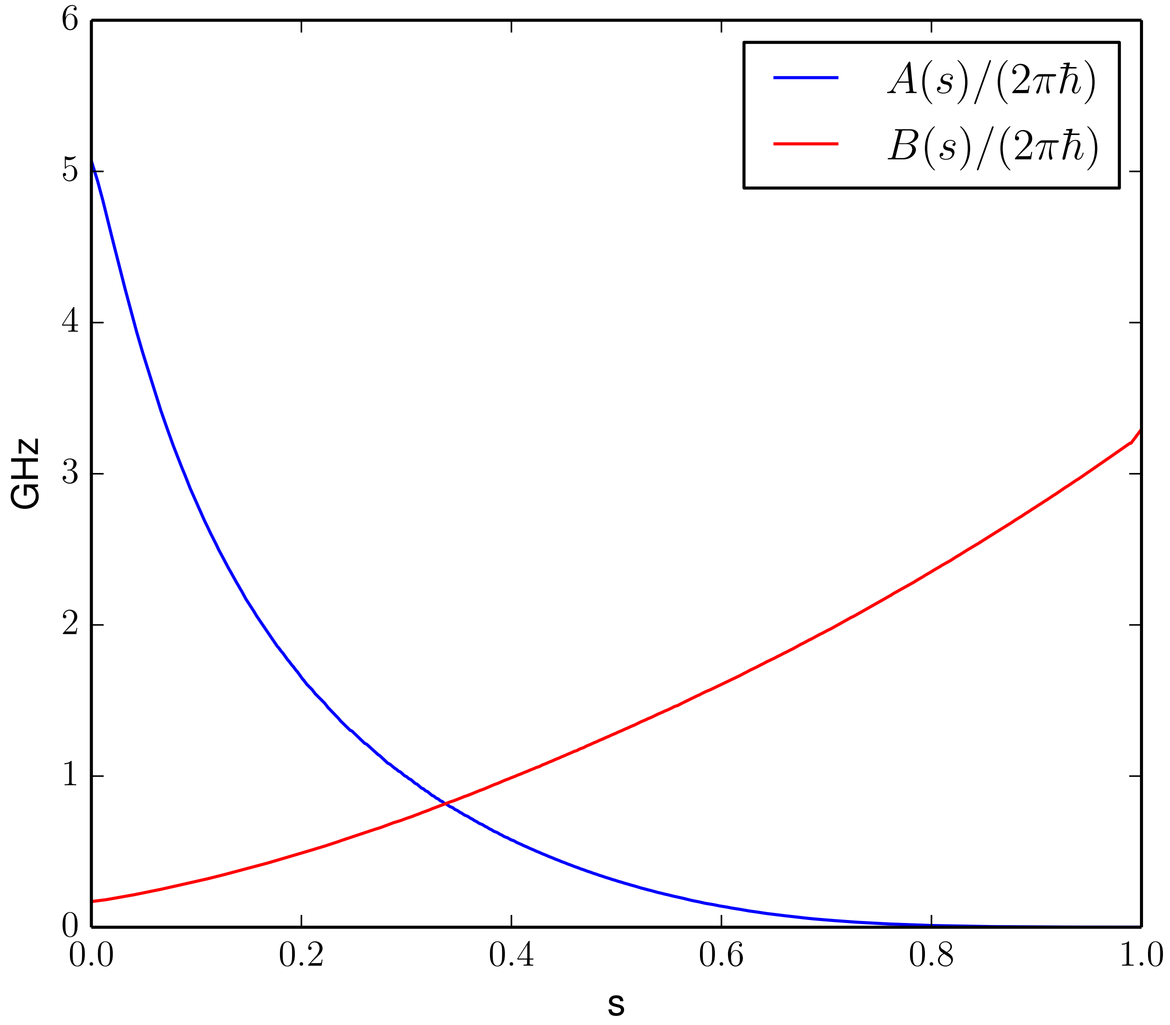

Supplementary Figure 5: **Annealing schedule.** Annealing energies $A(s)/(2\pi\hbar)$ and $B(s)/(2\pi\hbar)$ as a function of the annealing parameter s.

II. Supplementary Note 1

In this note we review the physics behind the flux qubits used in the D-Wave chip, and summarized all the relevant parameters for the device used in the experimental part of the paper.

A. Full Flux Qubit Hamiltonian

The full Compound Josephson Junction (CJJ) flux qubit Hamiltonian is better expressed in terms of flux phases, defined as renormalized fluxes $\phi = 2\pi\Phi/\Phi_0$ for any flux Φ. The Hamiltonian is [1]

$$\begin{aligned}\mathcal{H} = & - E_{\mathrm{C}}\partial_\phi^2 - E_{\mathrm{C_{CJJ}}}\partial^2_{\phi_{\mathrm{CJJ}}} + E_{\mathrm{J}}\cos(\phi)\cos(\phi_{\mathrm{CJJ}}/2) \\ & + E_{\mathrm{L}}\frac{(\phi-\phi^x)^2}{2} + E_{\mathrm{L_{CJJ}}}\frac{(\phi_{\mathrm{CJJ}}-\phi^x_{\mathrm{CJJ}})^2}{2} \ , \end{aligned} \quad (1)$$

where ϕ is the body flux phase to be quantized, ϕ^x is the external flux phase, ϕ_{CJJ} is the flux phase of the CJJ and ϕ^x_{CJJ} is the external flux of the CJJ (see Supplementary Figure 2). The energies of the different terms are given by

$$E_{\mathrm{C}} = \frac{(2e)^2}{2C} \qquad E_{\mathrm{C_{CJJ}}} = \frac{(2e)^2}{2(C/2)}$$
$$E_{\mathrm{J}} = \frac{I_c\Phi_0}{2\pi} \qquad E_{\mathrm{L}} = \left(\frac{\Phi_0}{2\pi}\right)^2\frac{1}{L+L_{\mathrm{CJJ}}/4}$$
$$E_{\mathrm{L_{CJJ}}} = \left(\frac{\Phi_0}{2\pi}\right)^2\frac{1}{L_{\mathrm{CJJ}}}$$

The parameters are the capacitance C, the body inductance of the main flux loop L and of the Compound Josephson Josephson L_{CJJ}, and the effective critical current of the Compound Josephson Junction I_c.

The median values for D-Wave's CJJ flux qubits in GHz are

$$\frac{E_{\mathrm{C}}}{2\pi\hbar} = 0.67 \text{ GHz} \qquad \frac{E_{\mathrm{C_{CJJ}}}}{2\pi\hbar} = 1.35 \text{ GHz}$$
$$\frac{E_{\mathrm{J}}}{2\pi\hbar} = 1071 \text{ GHz} \qquad \frac{E_{\mathrm{L}}}{2\pi\hbar} = 537 \text{ GHz}$$
$$\frac{E_{\mathrm{L_{CJJ}}}}{2\pi\hbar} = 11680 \text{ GHz} \ .$$

The CJJ flux phase ϕ^x_{CJJ} controls the quantum annealing evolution. The function $\phi^x_{\mathrm{CJJ}}(s)$ as a function of the annealing parameter for the quantum annealing schedule employed in this paper is plotted in Supplementary Figure 3.

Because $E_{\mathrm{L_{CJJ}}} \gg E_{\mathrm{L}}$ the phase ϕ_{CJJ} can be assumed to be centered at the value given by ϕ^x_{CJJ}, as a first approximation. The approximated flux qubit Hamiltonian is then

$$\begin{aligned}\mathcal{H}_s(\phi^x) = -E_{\mathrm{C}}\partial_\phi^2 + E_{\mathrm{J}}\cos(\phi)\cos(\phi^x_{\mathrm{CJJ}}(s)/2) \\ + E_{\mathrm{L}}\frac{(\phi-\phi^x)^2}{2} \ . \end{aligned} \quad (2)$$

This potential is plotted in Supplementary Figure 4a.

B. Effective qubit Hamiltonian

The effective qubit Hamiltonian is the simplified Hamiltonian $\mathcal{H}_s(\phi^x)$ of Eq. 2 projected into the two lowest energy energy levels $\{|\mathfrak{g}(s)\rangle, |\mathfrak{e}(s)\rangle\}$ of $\mathcal{H}_s(0)$

$$\mathcal{H}_s(\phi_x)\Big|_{\{|\mathfrak{g}(s)\rangle,|\mathfrak{e}(s)\rangle\}} = \mathcal{H}_s(0) + \phi^x\frac{\partial\mathcal{H}_s(0)}{\partial\phi^x}\Big|_{\{|\mathfrak{g}(s)\rangle,|\mathfrak{e}(s)\rangle\}} \ .$$

The eigenvectors of $\mathcal{H}_s(0)$ are symmetric and anti-symmetric superpositions of the flux up and down state in the double-well potential (see Supplementary Figure 4b)

$$|\mathfrak{g}(s)\rangle = \frac{1}{2}(|\uparrow(s)\rangle + |\downarrow(s)\rangle) \quad (3)$$
$$|\mathfrak{e}(s)\rangle = \frac{1}{2}(|\uparrow(s)\rangle - |\downarrow(s)\rangle) \ . \quad (4)$$

The gap between the ground state and the third excited state, depending on the annealing parameter s, goes between 10 and 8 GHz in the region of interest. This justifies the projection into the two lowest energy levels as long as the linear term in ϕ_x remains well below this energy.

Note that

$$\phi^x\frac{\partial\mathcal{H}_s(0)}{\partial\phi^x} = \Phi^x\frac{\Phi}{L+L_{\mathrm{CJJ}}/4} \ , \quad (5)$$

where Φ^x is the external flux and $\Phi/(L+L_{\mathrm{CJJ}}/4)$ is the persistent current operator. The eigenvectors of this operator are the flux up and down states $|\uparrow(s)\rangle, |\downarrow(s)\rangle$, with eigenvalues $\pm I_p(s)$. This defines the persistent current $I_p(s)$. We denote the gap between these states by $\Delta_1(s)$. In the basis of the up and down flux states we write

$$\begin{aligned}\mathcal{H}_s(0) + \phi^x\frac{\partial\mathcal{H}_s(0)}{\partial\phi^x}\Big|_{\{|\mathfrak{g}(s)\rangle,|\mathfrak{e}(s)\rangle\}} = \\ -\frac{1}{2}(\Delta_1(s)\sigma^x + \epsilon_1(\phi^x)\sigma^z) \ , \end{aligned} \quad (6)$$

where

$$\epsilon_1(\phi^x) = 2I_p(s)\Phi^x \quad (7)$$
$$\Phi/(L+L_{\mathrm{CJJ}}/4) = I_p(s)\sigma^z \ , \quad (8)$$

in this basis.

C. Coupling between qubits

The coupling between qubits has the form [2]

$$-J_{\mu\nu}E_{\mathrm{M}}(\phi_\mu - \phi^x_\mu)(\phi_\nu - \phi^x_\nu) \approx -J_{\mu\nu}E_{\mathrm{M}}\phi_\mu\phi_\nu \ , \quad (9)$$

where $J_{\mu\nu} \in [-1,1]$ is the dimensionless coupling. The corresponding energy is

$$\frac{E_{\mathrm{M}}}{2\pi\hbar} = \frac{1}{2\pi\hbar}\left(\frac{\Phi_0}{2\pi}\right)^2\frac{M_{\mathrm{AFM}}}{(L+L_{\mathrm{CJJ}}/4)^2} = 2.44 \text{ GHz} \ , \quad (10)$$

where in our case $M_{\rm AFM}$ is measured to be 1.41 pico henries.

In the two level qubit Hamiltonian approximation we use $\Phi/(L+L_{\rm CJJ}/4)\equiv I_p(s)\sigma^z$ to write

$$-J_{\mu\nu}E_{\rm M}\phi_\mu\phi_\nu \approx -J_{\mu\nu}M_{\rm AFM}I_p^2(s)\sigma^z_\mu\sigma^z_\nu = -B(s)J_{\mu\nu}\sigma^z_\mu\sigma^z_\nu$$

with the annealing function $B(s)$ defined as $B(s) = M_{\rm AFM}I_p^2(s)$. The superconducting flux qubits are calibrated so that $I_p(s)$ is the same for each of them.

D. External flux phase ϕ^x

The value of the external flux phase ϕ^x controls the strength of the local field in the single qubit Hamiltonian of Eq. 6. Note that $B(s)$, as defined above, scales with the persistent current squared. This is the reason why the external field flux is chosen (using our sign convention) as $\Phi^x = hM_{\rm AFM}I_p(s)$ so then (see Eq. 7)

$$\epsilon_1(\phi^x) = 2hM_{\rm AFM}I_p^2(s) = 2hB(s)\ . \tag{11}$$

Here $h\in[-1,1]$ is the dimensionless value of the local field as used in the main text. With this choice we write the annealing Hamiltonian as

$$\begin{aligned} H_0(s) &= -\frac{1}{2}\Delta_1(s)\sum_\mu \sigma^x_\mu - \frac{1}{2}\epsilon_1(\phi^x)\sigma^z_\mu - \sum_{\mu\nu} J_{\mu\nu}E_{\rm M}\phi_\mu\phi_\nu \\ &= -A(s)\sum_\mu \sigma^x_\mu - B(s)\left(\sum_\mu h_\mu\sigma^z_\mu + \sum_{\mu\nu}J_{\mu\nu}\sigma^z_\mu\sigma^z_\nu\right)\ . \end{aligned}$$

As mentioned above, the CJJ flux phase $\phi^x_{\rm CJJ}$ controls the quantum annealing evolution. Its value $\phi^x_{\rm CJJ}(s)$ plotted in Supplementary Figure 3 was chosen so that $I_p(s)$ scales linearly. In our case we have, for the Google-NASA D-Wave Two chip,

$$M_{\rm AFM}I_p(s)\frac{2\pi}{\Phi_0} \approx 10^{-3}(4.11s+1.21)\ . \tag{12}$$

The energy functions $A(s)$ and $B(s)$ are shown in Supplementary Figure 5.

E. Coupling to the bath

The interaction Hamiltonian of a single qubit with the bath is dominated by fluctuations on the flux body bias. The dimensional interaction Hamiltonian is

$$\mathcal{H}_{\rm SB} = \hat{I}\delta\Phi_x = \frac{\hat{\Phi}-\Phi_x}{L}\delta\Phi_x\ . \tag{13}$$

Projecting into the subspace $\{|\mathfrak{g}(s)\rangle, |\mathfrak{e}(s)\rangle\}$ as before we write (see Eq. 8)

$$\mathcal{H}_{\rm SB}(s) = I_p(s)\sigma^z\delta\Phi_x = \frac{1}{2}\sigma^z Q(s) \tag{14}$$

where

$$Q(s) = 2I_p(s)\delta\Phi_x\ . \tag{15}$$

The flux bias fluctuations are measured using microscopic resonant tunneling (MRT), as mentioned in the text. In particular MRT is performed at a point s with small tunneling amplitude $\Delta < 1$ MHz. Under these conditions we obtain the parameters for the noise spectral density $S_{\rm MRT}(\omega)$ which is defined in terms of a correlation function of the bath operators $Q(s)$ through the equation

$$S(\omega)_{\mu\nu} = \int_0^\infty dt e^{i\omega t}\langle e^{iH_Bt}Q_\mu e^{-iH_Bt}Q_\nu\rangle\ , \tag{16}$$

where μ and ν are qubit's indexes. From Eq. 15

$$\delta\Phi_x = \frac{Q_{\rm MRT}}{2I_p({\rm MRT})}\ , \tag{17}$$

which implies

$$Q(s) = \frac{I_p(s)}{I_p({\rm MRT})}Q_{\rm MRT} \approx \frac{I_p(s)}{I_p(1)}Q_{\rm MRT}\ . \tag{18}$$

This is the source of the dependence of the noise parameters on the annealing parameter, as mentioned in the text. The values corresponding to measurements done at the D-Wave Two chip for the noise parameters used in the NIBA Quantum Master Equation are

$$W_{\rm MRT}/(2\pi\hbar) = 0.4\,{\rm GHz}, \quad \eta_{\rm MRT} = 0.24\ . \tag{19}$$

III. Supplementary Note 2

In this note we give a more formal derivation of the effective potential used in the classical paths model [3, 4] using the Villain representation. We first introduce total spin operators for each unit cell (see Fig. 1a)

$$S_k^\alpha = \frac{1}{2}\sum_{j=1}^n \sigma^\alpha_{k,j}\ , \tag{20}$$

where $\alpha\in x,y,z$, and $k\in\{1,2\}$ denotes the left and right Chimera cells. It can be seen through numerical diagonalization that, because of the strong ferromagnetic couplings within each cell, we can obtain a good approximation to the exact quantum Hamiltonian using total spin operators. The approximate Hamiltonian is

$$\begin{aligned} H(s) \approx &- B(s)\left(\frac{1}{4}S_1^zS_2^z + \sum_{k=\{1,2\}}\left((S_k^z)^2 + 2h_kS_k^z\right)\right) \\ &- A(s)2\sum_k S_k^x\ . \end{aligned} \tag{21}$$

Next we introduce the Villain representation, which we use to derive a semiclassical Hamiltonian from large spin operators. In the spin basis $|M,S\rangle$ for total spin S, we introduce scaled spin operators $\mathfrak{s}^\alpha = S^\alpha/S$ for $\alpha = x,y,z$, and

$q = M/S$ an scaled quantum number. Denote $\epsilon = 1/S$ and

$$\mathfrak{s}^z|q\rangle = q|q\rangle \tag{22}$$

$$\mathfrak{s}^{\pm}|q\rangle = \sqrt{1+\epsilon - q(q\pm\epsilon)}|q\pm\epsilon\rangle \ . \tag{23}$$

We further introduce the canonically conjugated momentum operator $p = -i\,\epsilon\,\frac{\partial}{\partial q}$. The Villain representation in the limit of small ϵ (big n) is [3, 4]

$$\mathfrak{s}^{+} = e^{-ip}\sqrt{1+\epsilon - q(q+\epsilon)} \tag{24}$$

$$\mathfrak{s}^{-} = \sqrt{1+\epsilon - q(q+\epsilon)}e^{ip} \ . \tag{25}$$

These operators are Hermitian conjugates in this representation, and we will see that they have the correct action in the coordinate representations of the wave form

$$|\Psi\rangle = \int dq\ \Psi(q)|q\rangle \ . \tag{26}$$

We will use the property

$$e^{-\epsilon\frac{\partial}{\partial q}}F(q) = \sum_{a=0}^{\infty}\frac{(-\epsilon)^n}{n!}\frac{\partial^n}{\partial q^n}F(q) = F(q-\epsilon) \ . \tag{27}$$

We get

$$\mathfrak{s}^{+}|\Psi\rangle = \int dq\ e^{-\epsilon\frac{\partial}{\partial q}}\sqrt{1+\epsilon - q(q+\epsilon)}\ \Psi(q)|q\rangle \tag{28}$$

$$= \int dq\ \sqrt{1+\epsilon - (q-\epsilon)q}\ \Psi(q-\epsilon)|q\rangle \tag{29}$$

$$= \int dq\ \sqrt{1+\epsilon - q(q+\epsilon)}\ \Psi(q)|q+\epsilon\rangle \ , \tag{30}$$

and also

$$\mathfrak{s}^{-}|\Psi\rangle = \int dq\ \sqrt{1+\epsilon - q(q+\epsilon)}e^{\epsilon\frac{\partial}{\partial q}}\ \Psi(q)|q\rangle \tag{31}$$

$$= \int dq\ \sqrt{1+\epsilon - q(q+\epsilon)}\ \Psi(q+\epsilon)|q\rangle \tag{32}$$

$$= \int dq\ \sqrt{1+\epsilon - q(q-\epsilon)}\ \Psi(q)|q-\epsilon\rangle \tag{33}$$

Ignoring factors of order ϵ in Eqs. 24 and 25 we approximate

$$\mathfrak{s}^x = \frac{1}{2}(\mathfrak{s}^{+}+\mathfrak{s}^{-}) \approx \sqrt{1-q^2}\cos p \ . \tag{34}$$

Using the above representation, to the leading order in $1/n$ the Hamiltonian becomes

$$H^{\mathrm{WKB}}(q_1,q_2,p_1,p_2,s) = -nA(s)\sum_{k=1,2}\sqrt{1-q_k^2}\cos p_k$$
$$-nB(s)J\sum_{k=1,2}\left(h_k q_k + nq_k^2/4\right) - \frac{n}{2}B(s)J\,q_1\,q_2 \ .$$

The above Hamiltonian describes a pair of coupled ferromagnetic particles each with spin $n/2$. WKB theory based on this Hamiltonian describes eigenstates and eigenvalues with logarithmic accuracy in the asymptotic limit $n \gg 1$.

We can check the adequacy of the semiclassical Hamiltonian above even for $n = 8$ from the qualitative agreement of the gap of the original Hamiltonian and that obtained from the semiclassical Hamiltonian by the standard instanton method [3–5]. From the definition of the momentum operator $p = -i\,\epsilon\,\frac{\partial}{\partial q}$ we see that $\epsilon = 1/S$ plays the role of $\hbar$ in the WKB approximation, and we write the WKB ansatz for the semiclassical eigenstates $\Psi \propto \exp(W/\epsilon)$. The semiclassical Hamiltonian is

$$-m(q,t)(\cos p - 1) + V(q,t) \ , \tag{35}$$

where the effective q dependent mass is

$$m(q,t) = A(t)\sqrt{1-q^2}/\epsilon \ . \tag{36}$$

Using the instanton technique, the gap can be estimated as

$$R\exp\left(-\frac{1}{\epsilon}\int_{q_a}^{q_b} dq\,p(q)\right) \ , \tag{37}$$

where the exponent is the Euclidean action and $p(q)$ is the instanton trajectory between the double-well minima q_a and q_b. The instanton trajectory is obtained by going to imaginary time (mapping $p \to -ip$) and solving $p(q)$ in Eq. 35 to obtain

$$m(q,t)\,(1-\cosh p) + V(q,t) = V(q_a,t) \ . \tag{38}$$

We obtain

$$p(q) = \cosh^{-1}\left(\frac{V(q,t)-V(q_a,t)}{m(q,t)} + 1\right) \ . \tag{39}$$

For the WKB attempt rate R we use the separation between the first and third eigenstates of the quantum Hamiltonian, $R \approx 3$ GHz, as a proxy for the gap of the possible single well bound states. Plugging into Eq. 37, we obtain a sufficient qualitative agreement with the exact gaps

h_1	exact gap	instanton gap
0.48	10 MHz	5 MHz
0.47	36 MHz	33 MHz
0.46	78 MHz	85 MHz

(40)

The agreement improves for increasing n [3–5].

We can now consider the potential corresponding to a low energy description with very low momenta

$$U(q_1,q_2,s) = H^{\mathrm{WKB}}(q_1,q_2,0,0,s) \ . \tag{41}$$

The same potential is obtained in Ref. [5] approximating the projection of the Hamiltonian of large spin operators Eq. 21 over spin coherent states, which are product states. The effective potential in Fig. 1b in the main text corresponds to

$$\min_{q2} U(\cos\theta_1,q_2,s) \ . \tag{42}$$

Supplementary References

[1] Harris, R. *et al.* Experimental demonstration of a robust and scalable flux qubit. *Phys. Rev. B* **81**, 134510 (2010).

[2] Harris, R. *et al.* Compound josephson-junction coupler for flux qubits with minimal crosstalk. *Phys. Rev. B* **80**, 052506 (2009).

[3] Enz, M. & Schilling, R. Spin tunnelling in the semiclassical limit. *Journal of Physics C: Solid State Physics* **19**, 1765 (1986).

[4] Boulatov, A. & Smelyanskiy, V. N. Quantum adiabatic algorithm and large spin tunnelling. *Phys. Rev. A* **68** (2003).

[5] Farhi, E., Goldstone, J. & Gutmann, S. Quantum adiabatic evolution algorithms versus simulated annealing. *Preprint at http://arxiv.org/abs/quant-ph/0201031* (2002).
